# Shear-Induced Heteroaggregation of Oppositely Charged Colloidal Particles

**DOI:** 10.1021/acs.langmuir.0c01536

**Published:** 2020-08-19

**Authors:** Graziano Frungieri, Matthaus U. Babler, Marco Vanni

**Affiliations:** † Department of Applied Science and Technology, Politecnico di Torino, Corso Duca degli Abruzzi 24, 10129 Turin, Italy; ‡ Department of Chemical Engineering, KTH Royal Institute of Technology, Teknikringen 42, SE-10044 Stockholm, Sweden

## Abstract

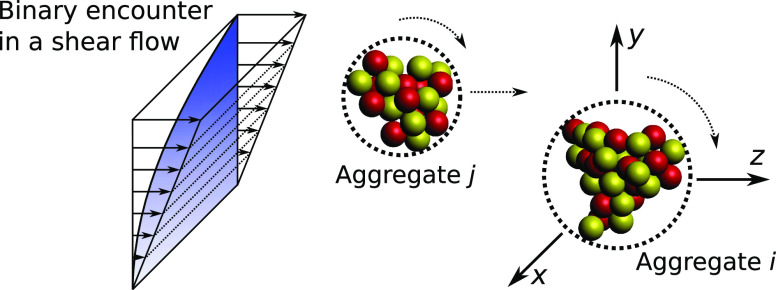

This paper investigates numerically the shear-induced aggregation of mixed populations of colloidal particles leading to the formation of clusters. Suspensions with different amounts of positively and negatively charged colloidal particles are simulated. To resolve the aggregation kinetics and structural properties of the formed clusters, we resort to a mixed deterministic-stochastic simulation method. The method is built on a combination of a Monte Carlo algorithm to sample a statistically expected sequence of encounter events between the suspended particles and a discrete element method built in the framework of Stokesian dynamics to simulate the encounters in a fully predictive manner. Results reveal a strong influence of the composition of the population on both the aggregation kinetics and the aggregate structure. In particular, we observe a size-stabilization phenomenon taking place in the suspension when the relative concentration of the majority particles lies in the range 80–85%; i.e., starting from primary particles, after a short growth period, we observed a cessation of aggregation. Inspection of the aggregate morphology shows that the formed clusters are composed of few minority particles placed in the inner region, while the aggregate surface is covered by majority particles, acting to provide a shielding effect against further growth.

## Introduction

Aggregation of colloidal particles is a relevant phenomenon encountered in a wide variety of natural and industrial settings, such as aquatic environments,^1,2^ wastewater treatment,^3^ drug and material syntheses,^4,5^ and food colloids.^6^ For instance, in wastewater treatment, it is common practice to induce aggregation of the suspended particles to form large clusters that can be more effectively removed from the dispersing medium by, e.g., sedimentation or filtration. However, dispersed particles frequently present a surface charge, which provides an energy barrier against aggregation. Therefore, to induce aggregation, the suspension must be destabilized. There exist a number of different methods to trigger the aggregation of charged particles:^7^ high-molecular-weight polymers can be used to promote the formation of bridges between particles;^8,9^ the increase in the ionic strength of the dispersing medium can be exploited to compress the electrical double layers and screen the electrostatic repulsion between the particles; and finally, the pH can be adjusted to neutralize the particle surface charge.^10−12^ When the increase in the solid content is of no importance to the outcome of the operation, the addition of particles bearing an opposite charge can be a viable option. The resulting aggregation process is often referred to as *heteroaggregation* in contrast to *homoaggregation*, i.e., the aggregation of colloidal particles of one single type.^13,14^

Although various studies addressing the heteroaggregation of oppositely charged particles are found in the literature,^14−100^ a full understanding of the heteroaggregation kinetics and aggregate structure is still missing. In fact, a number of parameters affect the phenomenon, namely, the nature of the solvent and the particles, the thickness of the electrical double layer, the relative concentration of anionic and cationic particles, the surface potentials of the particles, and the Péclet number characterizing the hydrodynamic environment experienced by the particles. This large parameter space makes a complete study of the problem extremely challenging.

At low Péclet number, the aggregation is driven by the Brownian motion of the particles. This aggregation mechanism is often referred to as *perikinetic* aggregation and has been extensively studied both numerically and experimentally. For instance, Kim et al.^14^ investigated the Brownian aggregation of a mixed population of oppositely charged particles with a small screening parameter. The study revealed that when interactions between particles are long-ranged, chainlike structures with the particle charge alternating down the chain are produced. López-López et al.^20^ performed diffusion-limited cluster–cluster aggregation simulations varying the relative amount of cationic and anionic particles. By imposing that only aggregation events between unlike particles can occur, they found a critical concentration separating two different aggregation regimes: when the fraction of particles of one type falls in the range 0.825–0.875, stable aggregates are produced. These aggregates are composed of few minority particles placed in the inner region of the aggregate, while the outer regions are populated by majority particles, thus providing a stabilization against further growth. For suspensions with a composition smaller than this critical concentration, they argue that such stabilization is absent and the aggregation proceeds indefinitely. A similar behavior was observed by AlSunaidi et al.^19^ who performed diffusion-limited cluster–cluster aggregation simulations. A slightly lower value for the critical concentration was found; however, this may be due to the geometric constraints imposed by the on-lattice approach used in their simulation.

When a flow field acts on the suspension, a different mechanism dominates the aggregation dynamics: starting from monomeric conditions, shear-driven aggregation is initially controlled by monomer–monomer aggregation. As soon as larger clusters are produced, the flow field acts by favoring the aggregation of these clusters as they present larger cross sections. This mechanism is generally referred to as *orthokinetic* aggregation and, for sufficiently large Péclet numbers, enhances the aggregation rate compared to a Brownian mechanism. Furthermore, as aggregates grow in size, the shear stresses exerted by the flow field on the aggregate structure induce substantial restructuring effects,^23,24^ thus altering the structure produced upon aggregation. For these reasons, if a critical concentration exists in *orthokinetic* heteroaggregation over which size stabilization occurs, its value may be different from the one characterizing *perikinetic* aggregation.

In this work, we study numerically the heteroaggregation of colloidal particles undergoing shear-induced aggregation. Next to exploring aggregation kinetics and structural characteristics of the formed aggregates, we in particular want to find out if the stabilization phenomena observed for Brownian aggregation also exist for shear aggregation. To this aim, we developed a mixed deterministic-stochastic numerical method able to simulate in detail the aggregation phenomena occurring in a sample volume of a colloidal suspension.^25^ Our numerical method is based on a combination of a Monte Carlo algorithm with a discrete element method built on Stokesian dynamics to properly account for hydrodynamic and colloidal interactions during an encounter between aggregates.^25−27^ The combination of these two computational methods provides profound insights both on the dynamics of the process and on the morphology of the aggregates. To keep the parameter space feasible, we focus on suspensions composed of cationic and anionic colloidal particles with small surface potentials and a large screening parameter to compare our work with earlier studies that focused on Brownian aggregation. Suspensions with different relative concentrations of cationic and anionic particles were simulated.

## Numerical Methods

We studied shear-induced aggregation in an aqueous suspension of spherical polystyrene colloidal particles with radius *a* = 500 nm. Particles bearing opposite surface charges with a low surface potential (Ψ = ±40 mV) and surrounded by a thin electrical double layer were considered. A dimensionless Debye screening parameter κ*a* = 50, with κ being the reciprocal of the Debye length, was adopted. A relatively mild shear rate γ̇ = 10 s^–1^ was assumed to act on the suspension. At room temperature, this set of parameters leads to a Péclet number  approximately equal to 6. In such conditions, it is reasonable to neglect the particle thermal motion and assume the encounters between the suspended particles to be driven uniquely by the gradient of the flow field. To study the effect of the shear rate, some simulations were run with a higher shear rate of γ̇ = 50 s^–1^, corresponding to *Pe* ≈ 30. We focused on the aggregation behavior of highly dilute colloidal suspensions. Under this circumstance, it is reasonable to reduce the aggregation dynamics to a sequence of binary encounter events, i.e., events that involve two aggregates at once. Based on this idea, we adopted a computational approach based on a combination of a Monte Carlo (MC) algorithm coupled to a discrete element method (DEM);^25^ the MC algorithm was used to sample a statistically expected sequence of binary encounter events, whereas the DEM was employed to simulate in detail the encounter between the sampled clusters. In fact, the DEM is able to rigorously account for all of the relevant forces acting on primary particles, namely, colloidal and hydrodynamic, allowing us to properly track their motion. The advantage of such a combination is the possibility to study a rich sample of a population of clusters with a reasonable computational effort while obtaining detailed information about the cluster morphology and the aggregation kinetics.

### Monte Carlo Algorithm

An event-driven, rejection-free Monte Carlo (MC) scheme was adopted to reproduce a particular realization of the aggregation process occurring in the suspensions. The scheme is based on the generation of random numbers obeying the same statistical laws governing the studied aggregation process. We started all of our simulations from monomeric conditions, i.e., the initial population is composed of 200 isolated particles dispersed in a suspension with a volume solid fraction φ = 10^–4^.

**Figure 1:**
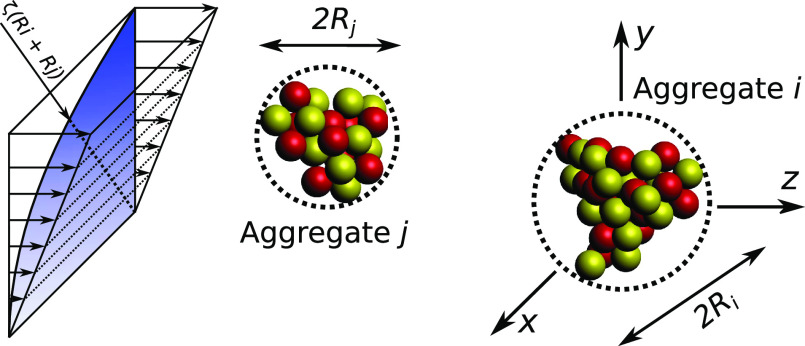
Typical configuration of an encounter event between a pair of aggregates in shear flow. The blue region represents a quarter of the encounter cross section.

To set up the MC algorithm, a model is needed to estimate the frequency of particle encounter, where by *encounter* we mean any event in which particles are driven into close proximity without necessarily colliding. To this purpose, we adapted the model developed by Smoluchowski for the two-body encounter kinetics.^28^ Although this model was derived based on the assumption that the involved particles are spheres that do not interact with each other and stick irreversibly upon contact, it is suitable to model the encounter frequency of aggregates after properly defining the encounter setup. Figure 1 depicts the typical configuration of an encounter involving a pair of aggregates in a shear flow of rate . The size of the aggregates is defined on the basis of their outer radius, i.e., the smallest radius *R* encompassing the whole aggregate structure. Encounters occur whenever an aggregate *j* passes close to an aggregate *i* after crossing the encounter cross section of radius ζ(*R*_*i*_ + *R*_*j*_), where the dimensionless parameter ζ is set equal to 1.2 in this work. Under these circumstances, the encounter frequency reads as
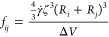
1where Δ*V* is the volume of the system in which the aggregates are suspended. Given the total number of suspended aggregates *N*_a_, the total encounter frequency can be computed as
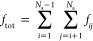
2From this piece of information, an interval of quiescence (IQ) can be estimated.^29^ The IQ has to be intended as a time interval during which no encounter occurs and the population remains unchanged. The following cumulative distribution function was adopted to sample stochastically the IQ
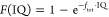
3The MC is also used to sample the aggregates involved in an encounter event: after computing the encounter frequencies relative to all of the possible binary encounters (eq 1), by picking a random number ξ from a uniform distribution between 0 and 1, the chosen event is the one with index *q* that satisfies the following relationship:
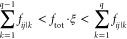
4where *q* can assume any value from 1 to *N*_a_(*N*_a_ – 1)/2, with *N*_a_ being the number of aggregates in the suspension.

Once a pair of aggregates has been picked, the MC is also in charge of selecting the initial coordinates of the aggregates in the flow field. Aggregate *i* is placed in the origin of the reference system, whereas the coordinate of the aggregate *j* is determined on the basis of statistical considerations. In a well-mixed suspension subject to a shear flow , the aggregate flux through the encounter cross section toward aggregate *i* increases linearly along the *y* direction and remains constant along the *x* direction; the *x*,*y*-coordinates of aggregate *j* are chosen to reproduce such distributions.^25^ The *z*_*j*_^0^-coordinate is set equal to ±(5*R*_*i*_ + *R*_*j*_) (with the ± sign determined according to the sign of the *y*-coordinate). At this distance, we can reasonably assume that the hydrodynamic interactions between the two aggregates are negligible and therefore the aggregates move with the same velocity of the undisturbed flow field. Furthermore, the two aggregates are given a random orientation about their center of mass, to take into account also the complex rotational motion they underwent before the encounter.

As a consequence of aggregation, the number of suspended particles decreases in time; to ensure the statistical robustness of our results, we adjusted the size of the simulated volume throughout the simulation: whenever the number of suspended aggregates fell below a threshold value (set equal to 3/4 of the initial number of suspended particles), the volume of the system was doubled and every aggregate cloned, thus preserving both the solid volume fraction and the particle size distribution.^30^

The MC algorithm presented here was tested for noninteracting particles (for which the encounter frequency of eq 1 is equal to the collision efficiency) and validated against the numerical solution of the Smoluchowski population balance equation. Further details can be found in refs (25, 31).

### DEM Simulation

The encounter event between two aggregates picked by the MC scheme is taken as the input to the DEM simulation, which allows for studying in detail the interactions of the aggregates during an encounter. The DEM was built in the framework of Stokesian dynamics (SD)^26^ to rigorously model the complete spectrum of the hydrodynamic interactions between primary particles, accounting for both the long-ranged and short-ranged interactions. Models for the colloidal interactions between primary particles were also included in our simulations.^25,27^

#### Colloidal Interaction

According to the Derjaguin–Landau–Verwey–Overbeek (DLVO) theory, the energy of interaction between two charged colloidal particles is expressed as the sum of two contributions
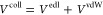
5where *V*^vdW^ is the energy associated with the van der Waals interaction and *V*^edl^ is the energy arising from the interaction between the electrical double layers of the particles. In this work, we used simple established models to compute in a pair additivity manner both kinds of interactions between primary particles.

The van der Waals attraction potential was modeled according to^32^
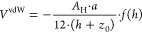
6where *h* represents the surface-to-surface distance between two interacting particles and *z*_0_ is the minimum approach distance, which is assumed in this work to be equal to *z*_0_ = 0.165 nm. Moreover, *A*_H_ is the composite Hamaker constant for the interaction of two solids immersed in a third medium, whereas the term *f*(*h*) is included to take into account the retardation effect, i.e., the steeper decrease of the intensity of the interaction between macroscopic bodies at large distances.^33^ It is worth pointing out that this interaction is of an attractive nature, regardless of the particle surface potential.

**Figure 2:**
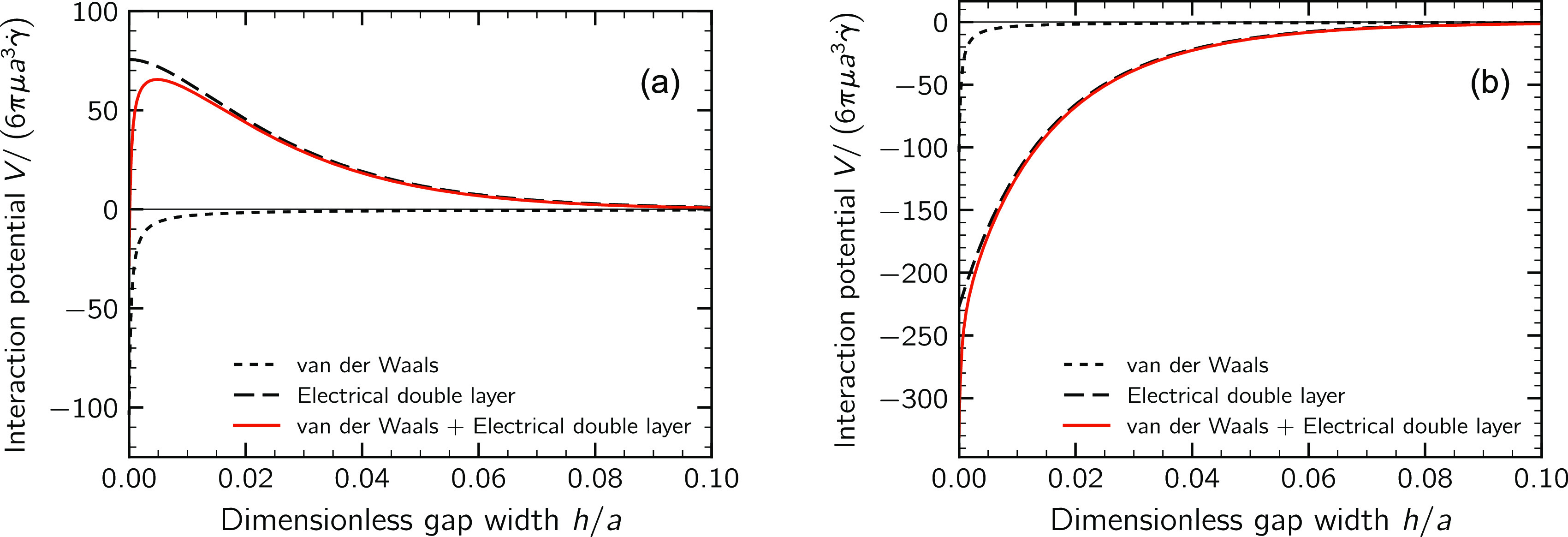
Interaction potential for a pair of primary particles with (a) same surface potential (Ψ_α_ = Ψ_β_ = 40 mV) and (b) opposite surface potentials (Ψ_α_ = −Ψ_β_ = 40 mV). For both cases, a dimensionless Debye screening parameter κ*a* = 50 was adopted.

The potential energy due to the interaction of the electrical double layers of two particles with constant surface potentials Ψ_α_ and Ψ_β_ was modeled as^34^
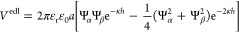
7where ε_0_ and ε_r_ represent, respectively, the vacuum permittivity and relative water permittivity, whereas κ represents the reciprocal Debye length. The energies of interaction for the cases of like and unlike particles are plotted in Figure 2, normalized with the convective energy provided by the shear flow field.

To avoid particle overlap, we also included in our DEM simulations a model to describe the contact mechanics in the presence of adhesion forces. A detailed description of the model can be found in refs (25) and (35).

It is finally worth pointing out that all of the forces are introduced in our model as normal forces, i.e., as forces acting along the line connecting the centers of two primary particles and no models accounting for the resistance to mutual sliding, rolling, and twisting motion were introduced.

#### Hydrodynamic Interaction

Hydrodynamic interactions between primary particles were modeled resorting to Stokesian dynamics in its force–torque–stresslet (FTS) formulation.^26^ This technique allowed us to relate the linear and angular velocities of a generic particle *p*(**u**_p_ and ***ω***_p_) to the hydrodynamic forces and torques (**F**_p_^H^ and **T**_p_^H^) acting on it by means of a set of linear equations of the form
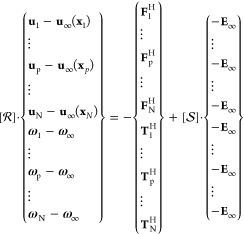
8where **u**_∞_(**x**_p_) is the velocity of the undisturbed velocity field evaluated at the center of particle *p*. In a shear flow **u**_∞_(**x**) = γ̇*y***e**_*z*_, with **e**_*z*_ being the unit vector aligned to the *z* direction, the undisturbed fluid angular velocity ***ω***_∞_ and the deformation rate tensor **E**_∞_ do not depend on the position **x** and their only nonzero elements are ω_∞,*x*_ = γ̇/2 and *E*_∞,*yz*_ = *E*_∞,*zy*_ = γ̇/2. The matrices  and , relating particle velocities to the hydrodynamic stresses, have dimensions 6*N* × 6*N* and 6*N* × 5*N*, respectively, with *N* being the total number of tracked primary particles; these matrices are generally referred to as resistance matrices and are built from two contributions: a far-field component obtained from a truncated multipole expansion of the flow field, which describes rigorously the interactions between particles when relatively far apart from each other, and a near-field correction, based on the lubrication theory, which is applied when particles are in close proximity. A cutoff length δ has been introduced to avoid the singularity of the lubrication force occurring when particles get in contact:^36,37^ when the gap *h* between particles becomes smaller than δ, the applied pair lubrication correction is no longer updated but kept evaluated at *h* = δ. In the simulations, we adopted δ = 0.1 nm.

In colloidal suspensions, the inertia of the particles is negligibly small compared to the other involved forces. Therefore, the linear system of eq 8 can be solved after imposing a simple force-torque balance of the following kind:
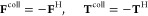
9where the colloidal force is calculated from the potential energy of colloidal interaction as , while the colloidal torque is zero. Once the linear and angular velocities **u**_p_ and ***ω***_p_ of the particles were obtained, an explicit Euler integration scheme with an adaptive time-step length was used to track the particle trajectories. A more exhaustive description of the method can be found in ref (25).

## Results and Discussion

### Aggregation of Primary Particles

The aim of this work is to study the aggregation occurring in suspensions in which particles with opposite surface potentials are dispersed in various relative amounts. Before discussing on the population aggregation dynamics, it is useful to investigate how primary particles aggregate depending on their surface potentials. In fact, the complex interplay between hydrodynamic and colloidal interactions determines the aggregation efficiency of colloidal particles,^38−41^ which, especially when starting from monomeric conditions, strongly affects the early stage of the aggregation process.

To evaluate aggregation cross sections and aggregation efficiencies, the discrete element method outlined in the previous section was combined with a grid-based technique.^42^ Given a pair of primary particles α, β with a common radius *a* equal to 500 nm and a shear flow field **u**_∞_ = γ̇*y***e**_*z*_ with γ̇ = 10 s^–1^, a 20 × 20 evenly spaced Cartesian grid was generated in a plane *z* = 10·*a* within the quadrant *y* > 0, *x* > 0.^38^ The size of each side of the grid was set equal to ζ(*a* + *a*), with ζ = 1.2. The setup of the encounter is comparable to the one depicted in Figure 1: at the beginning of each DEM simulation, the center of mass of the primary particle β was placed on a node of the grid, whereas particle α was placed in the origin of the reference system. From this initial configuration, the DEM was used to track the motion of both particles and to ascertain the outcome of the event, which can result in aggregation or missed aggregation: in the first case, the two particles collide generating a dimer, and in the second case, particles pass close to each other without colliding.

**Figure 3:**
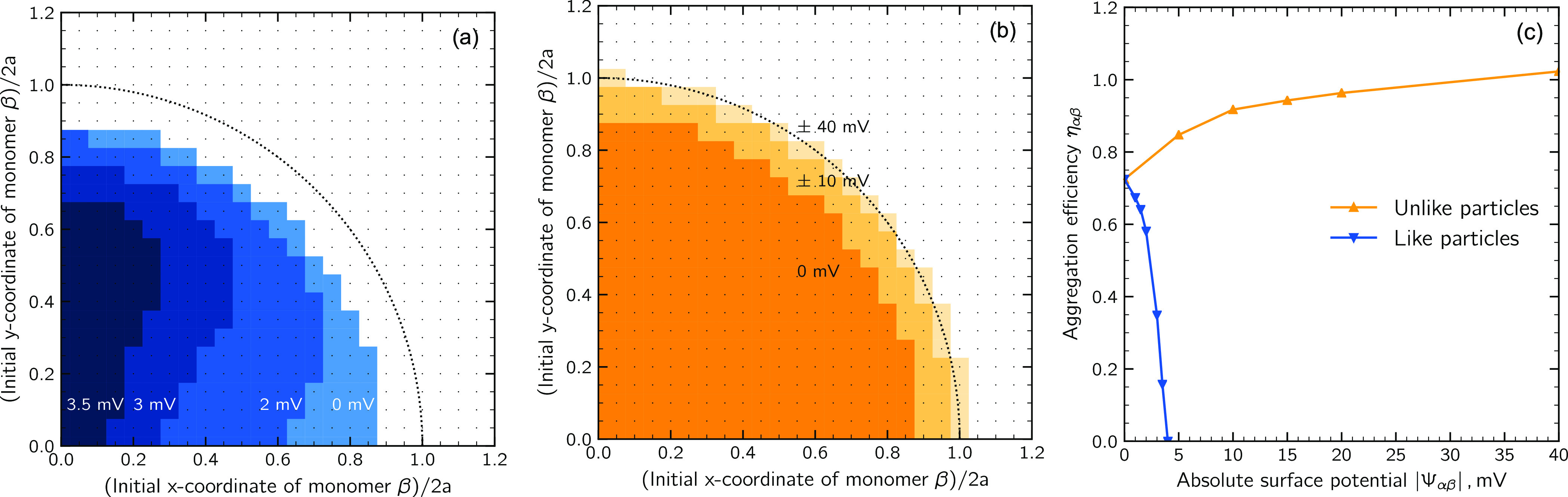
(a) Aggregation cross sections for a pair of like particles with surface potential ranging from 0 to 3.5 mV. (b) Aggregation cross sections for a pair of unlike particles with opposite surface potentials. (c) Aggregation efficiency as a function of the surface potentials of the particles.

Different values of the surface potential were considered. The obtained aggregation cross sections for the cases of like and unlike particles are depicted in Figure 3a and b, respectively. In both cases, |Ψ_α_| = |Ψ_β_|. For the aggregation of like particles, since the expression in eq 7 is symmetric with respect to the sign of the surface potential, only the case in which particles bear a positive surface charge was considered.

For aggregation of like particles (Figure 3a), the shape and size of the collision cross section are strongly dependent on the value of the common particle surface potential. For Ψ = 0 mV, a net short-ranged van der Waals attraction holds; however, the aggregation cross-section extension is significantly smaller than the one assumed by Smoluchowski (dotted curve in the plot). This is due to the hydrodynamic interaction between the particles, which prevents them from getting at a distance where the intensity of the van der Waals attraction is significant. Nevertheless, similar to the Smoluchowski cross section, the cross section found here exhibits a symmetric circular shape.^38,40^

When particles have a common surface potential Ψ > 0 mV, the cross sections become smaller in size and lose their symmetrical shape. This behavior is related to an interplay between colloidal and lubrication interactions and to the different range of action of electrical double layer and van der Waals interactions, with the latter being significantly more short-ranged; for the values of Ψ used herein, the total interaction *V*^coll^ is of an attractive nature at short distances and repulsive at larger distances.

Focusing on the cross section obtained for a common surface potential Ψ = 2 mV, it can be seen that the dimer formation probability changes according to the value of the initial *y*-coordinate of particle β. For low values of the initial *y*-coordinate, the relative approaching velocity between the particles is small and, as a consequence, the convective energy is not able to overcome the resistance to contact arising from both lubrication and electrical double-layer repulsion. As the initial *y*-coordinate is increased, particles approach each other faster and the convective energy succeeds in bringing the particles up to a distance in which attraction becomes important, prevailing over both lubrication and repulsive electrical double-layer interactions and thus leading to the aggregation of the particles. For even larger *y*-coordinates, the offset of the initial particle coordinates is significantly large and particles do not aggregate anymore. These arguments still hold when increasing the particle surface potentials. However, for larger Ψ, the aggregation cross sections become significantly smaller as a consequence of the increased intensity of the repulsive interaction and eventually aggregation no longer occurs for common surface potential Ψ > 4 mV. For such surface potentials, the electric double layer provides stabilization at the given shear rate. A qualitative similar behavior is observed when moderately increasing the shear rate, resulting in larger collision cross sections and a slightly larger value of the surface potential at which stabilization occurs.

On the contrary, when particles bear opposite surface charges, the extension of the aggregation cross section grows as the difference between their surface potential increases (Figure 3b). This behavior can be easily explained considering that more intense and more long-ranged attraction forces act on the particles when increasing their surface potentials.

From the simulations, the aggregation efficiency was calculated by computing the ratio between the flow rate of particles crossing the actual collision cross section *S*_act_ with the flow rate crossing the collision section assumed by the Smoluchowski model, circumscribed by a dotted curve in Figure 3a and b.^38,40^ Therefore, for a pair of primary particles α, β, the aggregation efficiency η_αβ_ reads as
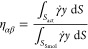
10Figure 3c reports the aggregation efficiency computed according to eq 10 as a function of the absolute surface potential. For the case of like particles, the aggregation efficiency goes rapidly to zero as the surface potentials increase over 4 mV. As already stated, this behavior is the consequence of the joint action of lubrication resistance and electrical double-layer repulsion, both acting to prevent particle aggregation. Conversely, for unlike particles, the aggregation efficiency shows a monotonic increasing trend as the difference between the surface potentials of the particle grows and assumes values slightly larger than 1 for Ψ = ±40 mV.

Finally, on the basis of the computed aggregation efficiencies, it is possible to reasonably conclude that for mixed populations of cationic and anionic particles with surface potentials equal to +40 mV, respectively, −40 mV, only heteroaggregation events can occur.

### Population Dynamics

The model system analyzed in this work is composed by neutrally buoyant, equally sized polystyrene particles with constant and opposite surface potentials (Ψ = ±40 mV), dispersed in a uniform shear flow (γ̇ = 10 s^–1^). The only parameter we varied in our simulations is the initial population composition; denoting with *A* and *B* the cationic and anionic primary particles, respectively, we can define the composition *x*_A_ as
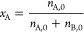
11where *n*_A,0_ and *n*_B,0_ indicate the initial number concentrations of primary particles A and B dispersed in the suspension, respectively. Because of the symmetry of the system, in which particles present the same absolute surface potentials, we studied only suspensions in which *x*_A_ ≥ 0.5. Therefore, in the following, A and B particles will also be referred to as majority and minority particles, respectively.

It is worth pointing out that we limit ourselves to the investigation of the initial stage of the process when only aggregation events take place, i.e., the aggregates never reached dimensions large enough to be vulnerable to breakage. The main parameters of the simulations are listed in Table 1.

**Table 1:** Physical Properties of the Simulated Suspensions

parameter	symbol	value
volume fraction of solid	φ	10^–4^
Hamaker constant	*A*_H_	0.97 × 10^–20^ J
particle surface potential	Ψ	±40 mV
reciprocal Debye length	κ	10^8^ m^–1^
vacuum permittivity	ε_0_	8.854 × 10^–12^ F m^–1^
water relative permittivity	ε_r_	80.1
minimum approach distance	*z*_0_	0.165 nm
monomer radius	*a*	500 nm
medium viscosity	μ	10^–3^ Pa s
shear rate	γ̇	10 s^–1^
population composition	*x*_A_	0.50–0.85

#### Early-Stage Kinetics

The early-stage kinetics of an aggregation process carried out starting from a population of isolated particles is determined almost exclusively by monomer–monomer aggregation events. As demonstrated in the previous section, with the adopted set of parameters, only heteroaggregation events involving oppositely charged particles can occur. Therefore, the rate of disappearance of monomer A, and equivalently of monomer B, can be described as
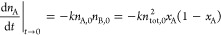
12where *k* represents the monomer aggregation rate given by the product of the Smoluchowski encounter rate (eq 1) with the aggregation efficiency η_AB_, and where *n*_tot,0_ is the initial total number concentration of both monomer A and monomer B.

**Figure 4:**
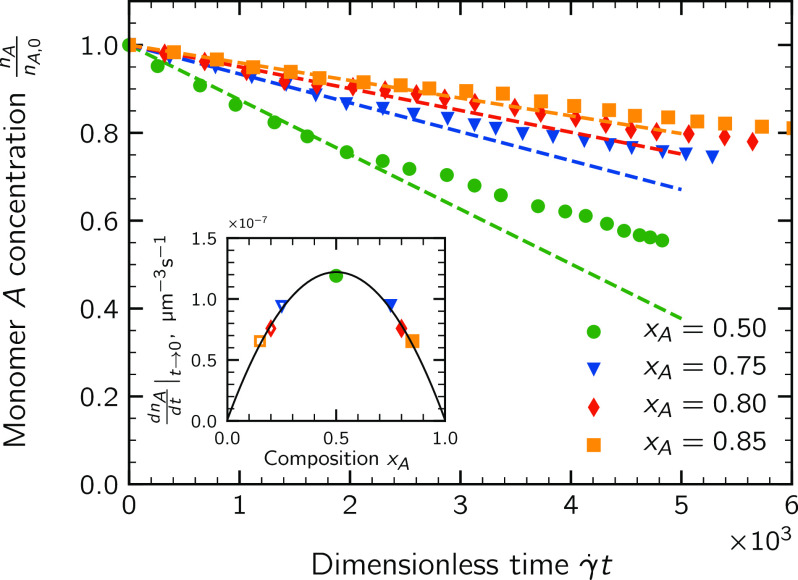
Time evolution of the normalized concentration of monomer A for the four suspensions analyzed. The symbols represent the simulation data obtained by averaging over five different realizations of the MC-DEM method. In the inset, the initial dimer formation rate is plotted as a function of the initial composition *x*_A_. Only simulations with *x*_A_ ≥ 0.5 were actually performed.

The main panel of Figure 4 reports the time evolution of the normalized number concentration of monomer A, *n*_A_/*n*_A,0_, obtained by averaging the simulation results over five different realizations of the MC-DEM method. The different data sets refer to different compositions of the suspensions, ranging from *x*_A_ = 0.50 to 0.85. The straight lines were obtained with a least-square fitting of the first 8 data points (corresponding to the first 80 encounters). As is apparent, regardless of the value of *x*_A_, initially the monomer A concentration follows fairly well a linearly decreasing trend. Deviations from the fitting lines appear only for larger times; this is due to the fact that as soon as dimers, trimers, and other larger clusters are produced, monomer–monomer encounters are no longer dominating the aggregation process. As inferable from the pair encounter frequency of eq 1, clusters, because of their larger cross section compared to that of monomers, are the most likely species to be involved in an aggregation event, thus causing a decrease in the rate of primary particle disappearance.

The values of the slopes of the straight lines are plotted in the inset of Figure 4 as a function of the composition *x*_A_ together with eq 12. It can be noticed that the computed values follow remarkably well the parabolic law of eq 12, represented by the continuous line, with the larger dimer formation rate observed in the symmetric system (*x*_A_ = 0.50). This can be easily explained considering that, when anionic and cationic particles are present in the suspension in the same amount, the probability for any pair of unlike particles to encounter each other in the suspension is at its maximum, but it sharply decreases as the suspension is enriched in one of the two classes of particles. This behavior confirms predictions by the HHF theory^18^ and observations by López-López et al.,^20^ who performed off-lattice Brownian dynamics simulations.

#### Late-Stage Kinetics

During the early stage, only aggregation events between monomers take place. This allowed us to derive a dimer formation rate constant by monitoring the time evolution of the monomer concentration. However, during the late stage, different events can occur, including both monomer–cluster and cluster–cluster aggregations. However, similar to the case of primary particles, encounters between clusters do not necessarily result in an aggregation; the aggregation efficiency is strongly dependent on the cluster morphology and surface composition, which may prevent or favor the aggregation. If the closest pairs of primary particles composing the two approaching clusters have an opposite surface potential, an aggregation event is likely to occur. On the contrary, if the aggregates approach each other with equally charged particles, once in close proximity, they may repel each other, thereby significantly deviating their trajectories and preventing the aggregates from colliding.

**Figure 5:**
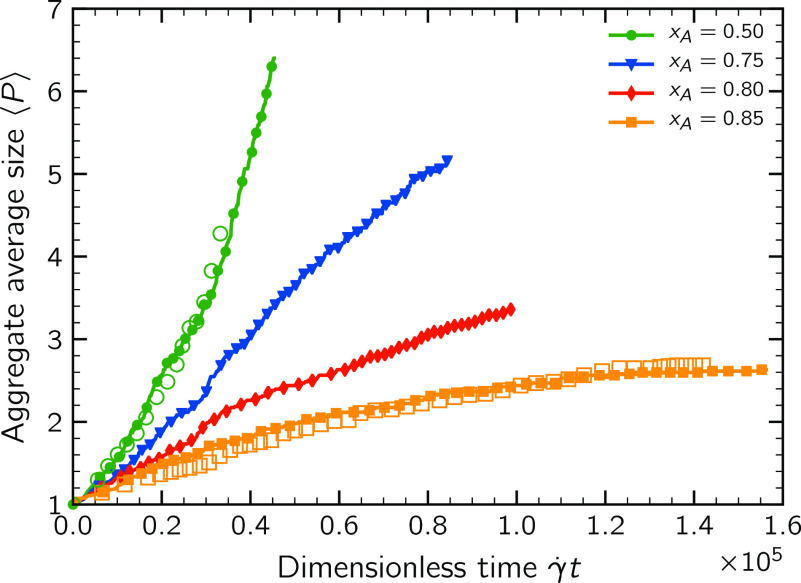
Time evolution of the average size of the suspended clusters expressed in terms of the average number of primary particles per aggregate ⟨*P*⟩. Solid symbols refer to the base case with γ̇ = 10 s^–1^, while empty symbols are for a higher shear rate γ̇ = 50 s^–1^.

To better understand how these phenomena affect the aggregation dynamics, it is useful to analyze the growth behavior of the suspensions. Figure 5 reports the temporal evolution of the cluster average size expressed in terms of number of constituent primary particles ⟨*P*⟩ for the four different initial compositions *x*_A_. All curves start from ⟨*P*⟩ = 1, corresponding to a monodisperse population of primary particles. As discussed in the previous section, the initial growth rate is strongly related to the composition of the population: it is the largest for the symmetric system (*x*_A_ = 0.50) and sharply reduces as the suspension is enriched in one of the two classes of particles. This phenomenon is still clearly visible in Figure 5. However, for longer times, a qualitatively different behavior emerges: for *x*_A_ = 0.50, the growth rate of the cluster average size increases throughout the aggregation process and the suspension reaches large values of ⟨*P*⟩ in a relatively short time. This behavior can be explained as the result of two distinct phenomena: first, during the initial stages, only one half of the monomer–monomer encounter can possibly lead to dimer formation. At a later stage, larger clusters composed of a comparable amount of anionic and cationic primary particles appear in this suspension. Therefore, during a binary encounter, even if clusters approach each other with a pair of like particles facing each other, the repulsive interaction may deviate their relative trajectories and a contact can still occur involving a different pair of primary particles. Second, the increased average cross section of the suspended clusters determines a speed-up of the growth process, with aggregation events that take place with an increased frequency.

For the population with *x*_A_ = 0.75, this self-accelerated growth dynamics is absent. As will be addressed in the next section, in this suspension, the clusters present a surface composition rather similar to the initial composition *x*_A_. For this reason, the probability of two unlike particles composing the approaching clusters to stick to each other is smaller compared to the symmetric system; thus, the cluster–cluster aggregation efficiency reduces substantially. This reduction is able to partially neutralize the speed-up we would expect as a result of the increased average cluster cross section, resulting in an almost linearly increasing average cluster size. A similar picture emerges from the suspension with composition *x*_*A*_ = 0.80.

Conversely, the growth behavior of the suspension *x*_A_ = 0.85 is substantially different: the cluster growth rate progressively slows down and for large times a plateau value of ⟨*P*⟩ is attained, meaning that the clusters stopped aggregating. A size stabilization took place in this suspension. These different trends suggest that similar to Brownian systems^19,20^ there also exists a critical initial composition *x*_A,c_ discriminating between the unlimited growth behavior and the size-stabilization phenomenon. Based on the growth kinetics, we can reasonably state that this critical initial composition falls in the range 0.80–0.85. For two population compositions, in the plot of Figure 5, the cluster growth kinetics for a larger shear rate (γ̇ = 50 s^–1^) is also reported. It can be seen that after normalizing the temporal scale with the shear rate intensity, the curves collapse one onto each other, thus showing that the effect of an increase of the shear rate reduces to an accelerated growth kinetics, with no relevant qualitative differences, at least for the range of values here investigated.

**Figure 6:**
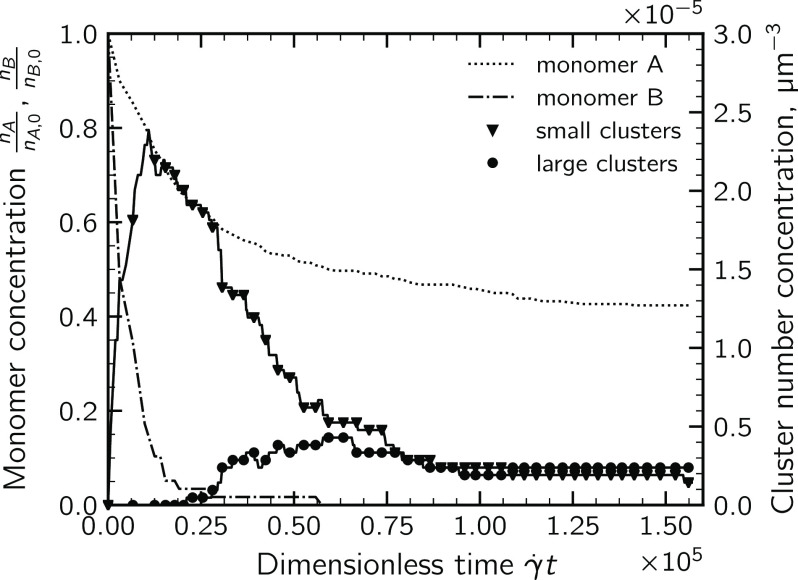
Temporal evolution of the particle size distribution for the suspension with composition *x*_A_ = 0.85. Please note the different scales of the left and right *y*-axis.

A deeper insight into the aggregation dynamics can be obtained by analyzing the temporal evolution of the particle size distribution. Figure 6 reports the time evolution of the concentration of the two classes of monomers together with the concentrations of large and small clusters. The latter are defined based on the number of constituent monomers: clusters composed of *P* ≥ 10 monomers are regarded as large ones, while clusters with 2 ≤ *P* < 10 are considered small. From the trends reported in the plot, it is possible to divide the aggregation dynamics into four subsequent steps.

1. The aggregation is dominated by monomer–monomer aggregation between unlike particles. As discussed in the previous section, this stage has a rather short duration.
2. Dimers and other small clusters appear in the suspension acting as growth seeds; they aggregate with themselves and with the monomers of both classes, which are still present in a significant amount in the suspension.
3. Minority particles B are totally consumed, but a significant amount of majority monomers A is still present. The aggregation is now dominated by monomer A–cluster and cluster–cluster aggregation. During this phase, clusters are progressively covered by majority monomers.
4. All superficial binding sites of the growing clusters are now saturated; clusters become stable, being fully covered by majority particles, and aggregation stops. The particle size distribution no longer changes in time, and an equilibrium state sets in the suspension. From the late-stage size distribution, one can notice that a significant amount of isolated majority monomers is still present in the suspension.

It is worth pointing out that the growth of clusters will be in any case limited by breakage phenomena. Once clusters attain a large-enough size, they become more vulnerable to breaking up as a consequence of the viscous stress exerted by the flow field. Therefore, in all of the suspensions in which a size-stabilization effect did not occur, a plateau value of ⟨*P*⟩ is expected to be reached at a later stage, as a consequence of an equilibrium between aggregation and breakage phenomena.^43−45^

### Cluster Characterization

**Figure 7:**
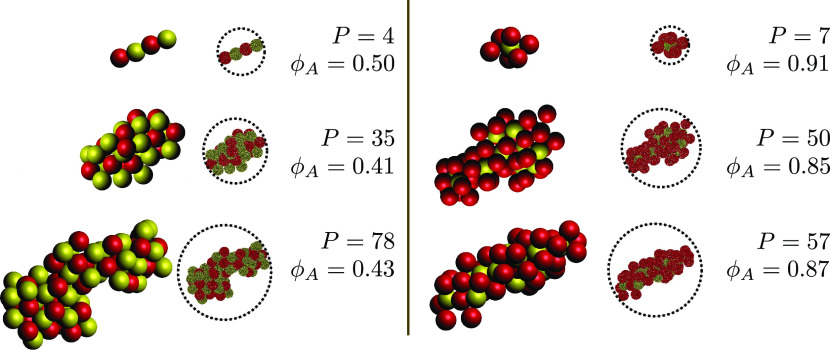
Representation of a sample of clusters from the populations *x*_A_ = 0.50 (left) and *x*_A_ = 0.85 (right): *P* is the number of constituent primary particles, and ϕ_A_ is a measure of their surface composition.

The long-time behavior of the suspensions can be better understood after characterizing the cluster structures produced upon aggregation. The aim of this characterization is to investigate how monomers are packed in clusters depending on the initial composition *x*_A_ of the suspension. To gain a deeper insight into the cluster morphology, the clusters produced in all of the four different suspensions were characterized by analyzing the cluster surface composition, the minority particle coordination number, and the average three-particle angle. Figure 7 shows a small sample of clusters together with some of the quantities used for the characterization. Notice that due to the relatively small size of the produced clusters, the fractal dimension could not be determined.

#### Surface Composition

The size-stabilization effect is related to the surface composition of the clusters produced upon aggregation. To quantitatively estimate the surface composition, a Monte Carlo mapping technique was developed: averaging over 100 randomly chosen orientations, the surface composition of the 2D projection of the clusters was evaluated by fictitiously hitting the clusters with 10^5^ darts whose coordinates were randomly sampled according to a uniform probability distribution spanning from −*R* to +*R*, with *R* being the radius of the circle encompassing the 2D projection of the cluster. The surface composition ϕ_A_ was then computed as the ratio between the number of darts that hit the monomer A and the total number of darts that hit the cluster projected area. Some of the berrylike representations obtained by such a technique are reported in Figure 7 together with the three-dimensional (3D) representations of the clusters.

**Figure 8:**
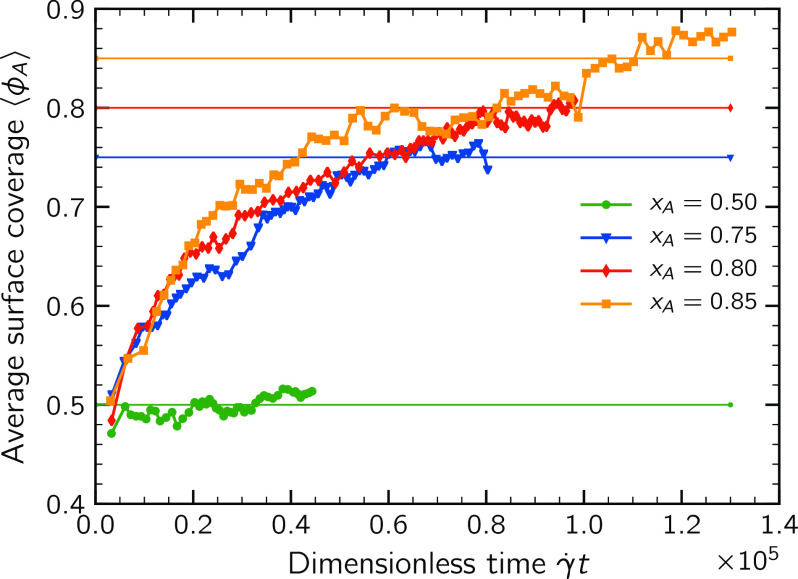
Average surface coverage of the populations of clusters as a function of time. Monomers were excluded from the calculation.

Figure 8 reports the average surface coverage of the four analyzed suspensions as a function of time. Monomers were excluded from the calculation, and the computation was started after the first 100 encounters took place. At this time, excluding monomers, the suspension is composed of mostly dimers and to a lesser extent of trimers and tetramers. All populations thus present an initial ⟨ϕ_A_⟩ equal approximately to 0.5. However, after a short time, the surface composition starts to differ significantly among the various populations. As predictable, in the symmetric system, ⟨ϕ_A_⟩ keeps oscillating around 0.50 throughout the aggregation, while the other systems show a rapidly increasing surface coverage. The rate of this increase has a weak dependence on the composition *x*_A_, but the most striking difference between the three systems emerges for large times, when an asymptotic value of ⟨ϕ_A_⟩ is attained: the suspensions *x*_A_ = 0.75 and 0.80 reach an asymptotic value that equals approximately the composition *x*_A_. On the contrary, for the suspension *x*_A_ = 0.85, a larger surface coverage was computed. This demonstrates that the size-stabilization effect taking place in this suspension is the result of a significant coverage of the outer surface of the clusters by majority particles.

Finally, it should be mentioned that the approach we adopted to evaluate ϕ_A_, even if it allowed us to get an accurate mapping of the surface of the clusters, fails in taking into account the hindrance effects: as it can be appreciated from the comparison between the 3D and the berrylike representations of the clusters depicted on the right-hand side of Figure 7, minority particles, even when placed in the most inner region of the aggregate and completely saturated by the bonded majority ones, are still detected by the Monte Carlo procedure we adopted.

#### Local Structure

**Figure 9:**
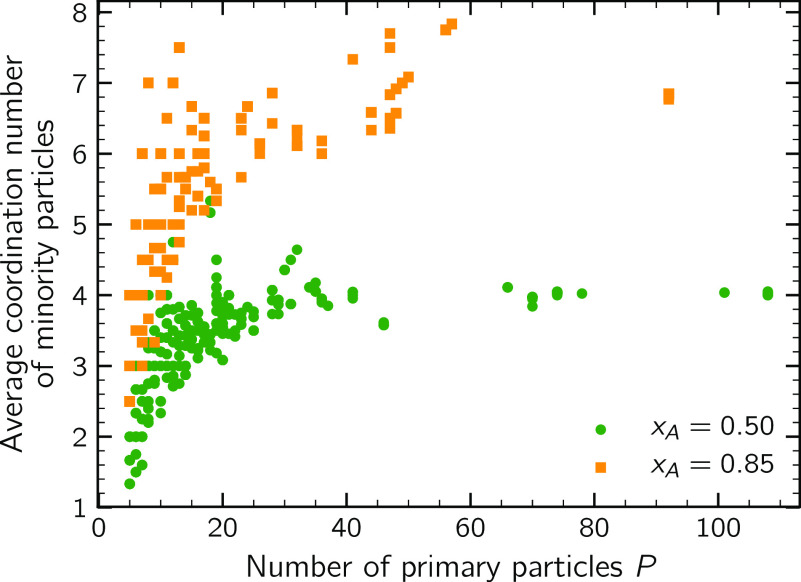
Average coordination number of minority particles as a function of the cluster size *P*. The data is obtained by sampling the growing suspension at a regular frequency of 100 encounters.

A quantitative characterization of the cluster morphology can be gained by looking at the average coordination *n*_c_ of the minority particles within the cluster. This quantity was computed averaging the values of the coordination number of the minority particles of each cluster. Figure 9 reports ⟨*n*_c_⟩ as a function of the cluster size. It emerges that ⟨*n*_c_⟩ shows a strong dependence on *x*_A_: the average coordination numbers of the suspension *x*_A_ = 0.85 are systematically larger than those of the suspension *x*_A_ = 0.50. This means that the primary particle packing of the clusters of these two populations is characterized by two distinct patterns. In the system *x*_A_ = 0.85, most of the minority particles are placed in the inner part of the cluster, while the outer parts of the cluster are populated by mainly majority particles. This behavior well compares with what was observed by Piechowiak et al.^15^ by both experimental and numerical investigations. Conversely, in the symmetric system, as it would be expected, there is a regular alternation of minority and majority particles throughout the structure. The lower average coordination number implies that a significant fraction of minority particles is placed on the surface of the clusters, exposed to the dispersing medium, and thus not fully saturated. Furthermore, it can be seen that for the symmetric system a well-defined plateau value of ⟨*n*_c_⟩ is attained, meaning that, as the cluster size increases, a self-similar monomer packing pattern establishes. On the contrary, in the suspension *x*_A_ = 0.85, the size stabilization that took place blocked the cluster growth before a clear self-similarity pattern set in.

From the values of coordination numbers, it is also inferable that significant restructuring effects take place after a contact establishes between a pair of unlike particles. In fact, the newly generated aggregates undergo a restructuring phenomenon in response to the shear stress, which leads to the creation of several new bonds. If that were not the case and progressively larger clusters were generated by the creation of one single new bond per aggregation event, we would have expected to obtain isostatic clusters with an average coordination number approaching 2 for sufficiently large sizes.^46^

Another useful characterization of the local cluster structure is given by the average three-particle angle ⟨θ⟩. To compute ⟨θ⟩, all of the existing groups of three connected primary particles formed by a minority particle (B) connected to two majority ones (A) were identified and characterized by the angle θ_ABA_′, formed by the two straight lines passing through the center of the intermediate particle B and through the centers of the two particles A. From this, the angle θ_ABA_′ was computed as
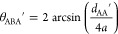
13where *d*_AA_′ represents the center-to-center distance between the two majority particles. This angle can vary between 60° and 180°, corresponding to an equilateral triangle and a linear chain, respectively. However, the first arrangement is hindered by the repulsive interaction between like particles A, which, because of the mutual repulsion they are subject to, always form angles larger than 60° with the central minority particle B.

**Figure 10:**
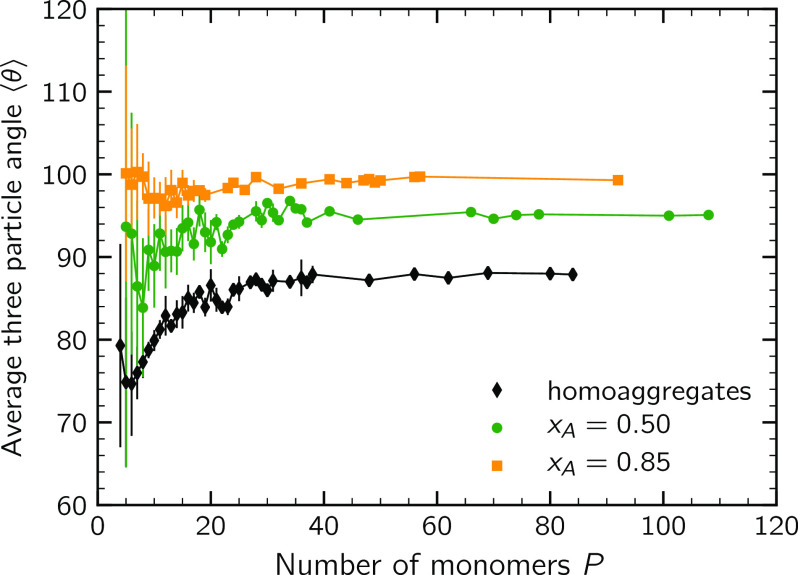
Average three-particle angle as a function of the cluster size. Error bars represent the standard deviation of the data. The plot also reports the data relative to homoaggregates from ref (25).

Figure 10 reports the average three-particle angle as a function of the cluster size. The data from the symmetric (*x*_A_ = 0.50) and the most enriched (*x*_A_ = 0.85) system is plotted together with the data obtained by the simulation of a homoaggregation process of a completely destabilized suspension.^25^ As noticeable, the average three-particle angles of the population *x*_A_ = 0.85 are systematically larger than the ones of the population *x*_A_ = 0.50. This implies that when there is a large excess of one kind of particles, the generated cluster presents a more open structure. This is due to the large number of majority particles, which arrange in such a way to avoid contact and minimize mutual repulsion, resulting in clusters with relatively large values of ⟨θ⟩. On the contrary, in the symmetric system, as a consequence of the regular alternation between the two kinds of monomers, the clusters are able to attain a more closely packed arrangement. As apparent, in homoaggregates, because of the purely attractive nature of the colloidal interactions, primary particles arrange in more compact structures resulting in smaller values of ⟨θ⟩.

## Conclusions

In the present work, we studied numerically the shear-induced aggregation occurring in dilute colloidal suspensions in which particles with opposite surface potentials are dispersed. We focused our attention on the relative concentration of the two types of particles and its effect on both the aggregation kinetics and cluster structure. To study a statistically significant population, a mixed stochastic-deterministic numerical method was employed. The method is based on a combination of a Monte Carlo algorithm employed to sample a statistically expected sequence of binary encounter events and a discrete element method used to ascertain in a fully predictive manner the outcome of each sampled event. The DEM was built in the framework of the Stokesian dynamic, to properly count hydrodynamic and colloidal interactions between particles.

The composition of the population emerged to have profound implications on both aggregation dynamics and cluster structures. Starting from monomeric conditions, the early-stage kinetics appeared to be strongly affected by the relative concentration of cationic and anionic particles: when they are present in an equal amount, the aggregation proceeds fast and a significant amount of dimers and other small clusters is promptly produced. On the contrary, when the suspension is enriched in one of the two classes of particles, the rate of dimer formation is substantially reduced. A simple model based on reaction stoichiometry was proposed and showed to properly fit the simulation data.

Furthermore, the late-stage kinetics showed substantial qualitative differences depending on the population composition. In the symmetric system, the growth dynamics showed a self-accelerating behavior, with clusters that soon reached quite large sizes. In the system in which the initial population was formed by 85% of one kind of particles, the aggregation rate progressively slowed down and, for sufficiently large times, a size stabilization took place, i.e., stable aggregates appeared in the suspension. These aggregates were formed by a core in which particles of the two classes are both present and by an external shell fully covered by majority particles, thus providing a shielding effect against further aggregation. Finally, the characterization of the internal structure of the aggregates showed that different monomer packing patterns have to be expected in mixed populations of oppositely charged particles.

## References

[ref1] Praetorius A.; Badetti E.; Brunelli A.; Clavier A.; Gallego-Urrea J. A.; Gondikas A.; Hassellöv M.; Hofmann T.; Mackevica A.; Marcomini A.; Peijnenburg W.; Quik J. T. K.; Seijo M.; Stoll S.; Tepe N.; Walch H.; Von der Kammer F. Strategies for determining heteroaggregation attachment efficiencies of engineered nanoparticles in aquatic environments. Environ. Sci.: Nano 2020, 7, 351–367. 10.1039/c9en01016e.

[ref2] Oriekhova O.; Stoll S. Heteroaggregation of nanoplastic particles in the presence of inorganic colloids and natural organic matter. Environ. Sci.: Nano 2018, 5, 792–799. 10.1039/C7EN01119A.

[ref3] Rhein F.; Scholl F.; Nirschl H. Magnetic seeded filtration for the separation of fine polymer particles from dilute suspensions: Microplastics. Chem. Eng. Sci. 2019, 207, 1278–1287. 10.1016/j.ces.2019.07.052.

[ref4] Arpagaus C. PLA/PLGA nanoparticles prepared by nano spray drying. J. Pharm. Invest. 2019, 405–426. 10.1007/s40005-019-00441-3.

[ref5] Cerbelaud M.; Videcoq A.; Rossignol F.; Piechowiak M. A.; Bochicchio D.; Ferrando R. Heteroaggregation of ceramic colloids in suspensions. Adv. Phys.: X 2017, 2, 35–53. 10.1080/23746149.2016.1254064.

[ref6] Mao Y.; McClements D. J. Modulation of food texture using controlled heteroaggregation of lipid droplets: principles and applications. J. Appl. Polym. Sci. 2013, 130, 3833–3841. 10.1002/app.39631.

[ref7] Yates P. D.; Franks G. V.; Jameson G. J. Orthokinetic heteroaggregation with nanoparticles: effect of particle size ratio on aggregate properties. Colloids Surf., A 2008, 326, 83–91. 10.1016/j.colsurfa.2008.05.030.

[ref8] Biggs S.; Habgood M.; Jameson G. J.; Yan Y. D. Aggregate structures formed via a bridging flocculation mechanism. Chem. Eng. J. 2000, 80, 13–22. 10.1016/S1383-5866(00)00072-1.

[ref9] Zhou Y.; Franks G. V. Flocculation mechanism induced by cationic polymers investigated by light scattering. Langmuir 2006, 22, 6775–6786. 10.1021/la060281+. 16863222

[ref10] Jiang J.; Oberdörster G.; Biswas P. Characterization of size, surface charge, and agglomeration state of nanoparticle dispersions for toxicological studies. J. Nanopart. Res. 2009, 11, 77–89. 10.1007/s11051-008-9446-4.

[ref11] Liu W.; Sun W.; Borthwick A. G. L.; Ni J. Comparison on aggregation and sedimentation of titanium dioxide, titanate nanotubes and titanate nanotubes-TiO2: Influence of pH, ionic strength and natural organic matter. Colloids Surf., A 2013, 434, 319–328. 10.1016/j.colsurfa.2013.05.010.

[ref12] Lattuada M.; Hatton T. A. Preparation and controlled self-assembly of Janus magnetic nanoparticles. J. Am. Chem. Soc. 2007, 129, 12878–12889. 10.1021/ja0740521. 17910450

[ref13] Kim A. Y.; Berg J. C. Fractal heteroaggregation of oppositely charged colloids. J. Colloid Interface Sci. 2000, 229, 607–614. 10.1006/jcis.2000.7028. 10985842

[ref14] Kim A. Y.; Hauch K. D.; Berg J. C.; Martin J. E.; Anderson R. A. Linear chains and chain-like fractals from electrostatic heteroaggregation. J. Colloid Interface Sci. 2003, 260, 149–159. 10.1016/S0021-9797(03)00033-X. 12742045

[ref15] Piechowiak M. A.; Videcoq A.; Rossignol F.; Pagnoux C.; Carrion C.; Cerbelaud M.; Ferrando R. Oppositely charged model ceramic colloids: numerical predictions and experimental observations by confocal laser scanning microscopy. Langmuir 2010, 26, 12540–12547. 10.1021/la101027d. 20604541

[ref16] Cerbelaud M.; Ferrando R.; Videcoq A. Simulations of heteroaggregation in a suspension of alumina and silica particles: effect of dilution. J. Chem. Phys. 2010, 132, 084701 10.1063/1.3328876. 20192311

[ref17] Puertas A. M.; Fernández-Barbero A.; de las Nieves F. J. Kinetics of colloidal heteroaggregation. Physica A 2002, 304, 340–354. 10.1016/S0378-4371(01)00564-7.

[ref18] Hogg R.; Healy T. W.; Fuerstenau D. W. Mutual coagulation of colloidal dispersions. Trans. Faraday Soc. 1966, 62, 1638–1651. 10.1039/tf9666201638.

[ref19] AlSunaidi A.; Lach-Hab M.; González A. E.; Blaisten-Barojas E. Cluster-cluster aggregation in binary mixtures. Phys. Rev. E 2000, 61, 550 10.1103/PhysRevE.61.550. 11046296

[ref20] López-López J. M.; Moncho-Jordá A.; Schmitt A.; Hidalgo-Álvarez R. Formation and structure of stable aggregates in binary diffusion-limited cluster-cluster aggregation processes. Phys. Rev. E 2005, 72, 031401 10.1103/PhysRevE.72.031401. 16241429

[ref21] Okuzono T.; Odai K.; Masuda T.; Toyotama A.; Yamanaka J. Numerical study of cluster formation in binary charged colloids. Phys. Rev. E 2016, 94, 012609 10.1103/PhysRevE.94.012609. 27575181

[ref22] Lattuada M.; Morbidelli M. Effect of repulsive interactions on the rate of doublet formation of colloidal nanoparticles in the presence of convective transport. J. Colloid Interface Sci. 2011, 355, 42–53. 10.1016/j.jcis.2010.11.070. 21193203

[ref100] Zaccone A.; Wu H.; Gentili D.; Morbidelli M. Theory of activated-rate processes under shear with application to shear-induced aggregation of colloids. Phys. Rev. E 2009, 80, 051404. 10.1103/PhysRevE.80.051404 20364982

[ref23] Becker V.; Schlauch E.; Behr M.; Briesen H. Restructuring of colloidal aggregates in shear flows and limitations of the free-draining approximation. J. Colloid Interface Sci. 2009, 339, 362–372. 10.1016/j.jcis.2009.07.022. 19726052

[ref24] Ren Z.; Harshe Y. M.; Lattuada M. Influence of the potential well on the breakage rate of colloidal aggregates in simple shear and uniaxial extensional flows. Langmuir 2015, 31, 5712–5721. 10.1021/la504966y. 25941836

[ref25] Frungieri G.; Vanni M. Shear-induced aggregation of colloidal particles: A comparison between two different approaches to the modelling of colloidal interactions. Can. J. Chem. Eng. 2017, 95, 1768–1780. 10.1002/cjce.22843.

[ref26] Brady J. F.; Bossis G. Stokesian dynamics. Annu. Rev. Fluid Mech. 1988, 20, 111–157. 10.1146/annurev.fl.20.010188.000551.

[ref27] Seto R.; Botet R.; Auernhammer G. K.; Briesen H. Restructuring of colloidal aggregates in shear flow. Eur. Phys. J. E 2012, 35, 128 10.1140/epje/i2012-12128-4. 23229757

[ref28] Smoluchowski M. A mathematical theory of coagulation kinetics of colloidal solutions. Z. Phys. Chem. 1917, 92, 192.

[ref29] Shah B. H.; Ramkrishna D.; Borwanker J. D. Simulation of particulate systems using the concept of the interval of quiescence. AIChE J. 1977, 23, 897–904. 10.1002/aic.690230617.

[ref30] Liffman K. A direct simulation Monte-Carlo method for cluster coagulation. J. Comput. Phys. 1992, 100, 116–127. 10.1016/0021-9991(92)90314-O.

[ref31] Frungieri G. A Novel Monte Carlo - Discrete Element Method Approach for the Micro-Mechanics of Colloidal Suspensions. Ph.D. thesis, Politecnico di Torino, 2018.

[ref32] Hamaker H. C. The London—van der Waals attraction between spherical particles. Physica 1937, 4, 1058–1072. 10.1016/S0031-8914(37)80203-7.

[ref33] Wiese G. R.; Healy T. W. Effect of particle size on colloid stability. Trans. Faraday Soc. 1970, 66, 490–499. 10.1039/tf9706600490.

[ref34] Ohshima H. Electrostatic interaction between two dissimilar spheres: An explicit analytic expression. J. Colloid Interface Sci. 1994, 162, 487–495. 10.1006/jcis.1994.1064.

[ref35] Johnson K. L.; Kendall K.; Roberts A. D. Surface energy and the contact of elastic solids. Proc. R. Soc. London, Ser. A 1971, 324, 301–313. 10.1098/rspa.1971.0141.

[ref36] Seto R.; Mari R.; Morris J. F.; Denn M. M. Discontinuous shear thickening of frictional hard-sphere suspensions. Phys. Rev. Lett. 2013, 111, 218301 10.1103/PhysRevLett.111.218301. 24313532

[ref37] Trulsson M.; Andreotti B.; Claudin P. Transition from the viscous to inertial regime in dense suspensions. Phys. Rev. Lett. 2012, 109, 118305 10.1103/PhysRevLett.109.118305. 23005688

[ref38] Vanni M.; Baldi G. Coagulation efficiency of colloidal particles in shear flow. Adv. Colloid Interface Sci. 2002, 97, 151–177. 10.1016/S0001-8686(01)00050-1. 12027019

[ref39] van de Ven T. G. M.; Mason S. G. The microrheology of colloidal dispersions: IV. Pairs of interacting spheres in shear flow. J. Colloid Interface Sci. 1976, 57, 505–516. 10.1016/0021-9797(76)90229-0.

[ref40] van de Ven T. G. M.; Mason S. G. The microrheology of colloidal dispersions VII. Orthokinetic doublet formation of spheres. Colloid. Polym. Sci. 1977, 255, 468–479. 10.1007/BF01536463.

[ref41] Kroll-Rabotin J.-S.; Gisselbrecht M.; Ott B.; May R.; Fröhlich J.; Bellot J.-P. Multiscale Simulation of Non-Metallic Inclusion Aggregation in a Fully Resolved Bubble Swarm in Liquid Steel. Metals 2020, 10, 517 10.3390/met10040517.

[ref42] Frungieri G.; Vanni M. In Dynamics of a Shear-Induced Aggregation Process by a Combined Monte Carlo-Stokesian Dynamics Approach, Proceedings of the 9th International Conference on Multiphase Flow, May 22–27, 2016, Firenze, Italy.

[ref43] Vanni M. Approximate population balance equations for aggregation-breakage processes. J. Colloid Interface Sci. 2000, 221, 143–160. 10.1006/jcis.1999.6571. 10631014

[ref44] Sadegh-Vaziri R.; Ludwig K.; Sundmacher K.; Babler M. U. Mechanisms behind overshoots in mean cluster size profiles in aggregation-breakup processes. J. Colloid Interface Sci. 2018, 528, 336–348. 10.1016/j.jcis.2018.05.064. 29885609

[ref45] Frungieri G.; Boccardo G.; Buffo A.; Marchisio D.; Karimi-Varzaneh H. A.; Vanni M. A CFD-DEM approach to study the breakup of fractal agglomerates in an internal mixer. Can J. Chem. Eng. 2020, 98, 1880–1892. 10.1002/cjce.23773.

[ref46] Gastaldi A.; Vanni M. The distribution of stresses in rigid fractal-like aggregates in a uniform flow field. J. Colloid Interface Sci. 2011, 357, 18–30. 10.1016/j.jcis.2011.01.080. 21333303

